# Galectins in Early Pregnancy and Pregnancy-Associated Pathologies

**DOI:** 10.3390/ijms23010069

**Published:** 2021-12-22

**Authors:** Milica Jovanović Krivokuća, Aleksandra Vilotić, Mirjana Nacka-Aleksić, Andrea Pirković, Danica Ćujić, Janko Legner, Dragana Dekanski, Žanka Bojić-Trbojević

**Affiliations:** Institute for Application of Nuclear Energy Department for Biology of Reproduction, University of Belgrade, Banatska 31b, 11080 Belgrade, Serbia; milicaj@inep.co.rs (M.J.K.); aleksandrav@inep.co.rs (A.V.); mnacka@inep.co.rs (M.N.-A.); andrea.pirkovic@inep.co.rs (A.P.); danicac@inep.co.rs (D.Ć.); janko.legner@inep.co.rs (J.L.); dragana.dekanski@inep.co.rs (D.D.)

**Keywords:** galectins, trophoblast function, pregnancy, pregnancy-related pathologies

## Abstract

Galectins are a family of conserved soluble proteins defined by an affinity for β-galactoside structures present on various glycoconjugates. Over the past few decades, galectins have been recognized as important factors for successful implantation and maintenance of pregnancy. An increasing number of studies have demonstrated their involvement in trophoblast cell function and placental development. In addition, several lines of evidence suggest their important roles in feto-maternal immune tolerance regulation and angiogenesis. Changed or dysregulated galectin expression is also described in pregnancy-related disorders. Although the data regarding galectins’ clinical relevance are still at an early stage, evidence suggests that some galectin family members are promising candidates for better understanding pregnancy-related pathologies, as well as predicting biomarkers. In this review, we aim to summarize current knowledge of galectins in early pregnancy as well as in pregnancy-related pathologies.

## 1. Introduction

Pregnancy outcome depends on complex and tightly regulated mechanisms which occur on both sides of the feto-maternal interface. It is well documented that successful pregnancy is a result of several steps, including maternal immune adaptation, development of implantation competent blastocyst, implantation to a prepared endometrium and development of functional placenta. In the first trimester of pregnancy, trophoblast cells in human placenta undergo differentiation in two pathways—villous and extravillous. By acquiring a migratory and invasive phenotype, extravillous trophoblast cells (EVT) subsequently invade maternal decidua and transform uterine spiral arteries. Accumulated data have shown that various molecules influence trophoblast cell migration and invasion [1]. Complex crosstalk between integrin cell receptors and extracellular matrix (ECM) components is of particular importance for trophoblast function. In addition to classic ECM molecules, proteins such as galectins have been recognized to be necessary for the establishment and maintenance of pregnancy [2,3,4].

Galectins are defined by an affinity for β-galactoside structures present on glycoconjugates. This interaction is mediated via carbohydrate recognition domain (CRD), which possesses significant structural similarity among family members [5]. Thus far, at least 13 of 19 galectin family members have been identified in humans, displaying different intra- and extracellular localizations and biological functions [6]. Through preferential recognition of N-acetyllactosamine residues present in diverse cell surface and ECM glycans, galectins exert functions dependent on their lectin activity [5]. This characteristic enables galectins to translate information present in glycocodes to certain cellular functions. An important feature of galectins is their ability to act inside the cells mainly through protein–protein interactions, affecting apoptosis, cell cycle and pre-mRNA splicing [7]. In line with this multifunctionality, altered expression and/or function of galectins has often been associated with various pathologies, suggesting their potential use as biomarkers.

Over the past few decades, galectins have been recognized as contributing factors in reproductive processes, including blastocyst implantation, feto-maternal immune tolerance, placental development and angiogenesis. Although the majority of these studies focused on galectin-1 and -3, involvement of other family members in pregnancy orchestration elicits great interest. We aim to summarize in this review the current knowledge of galectins in early pregnancy as well as in pregnancy-related pathologies. 

## 2. Galectin Expression Pattern in Placenta

**Figure 1:**
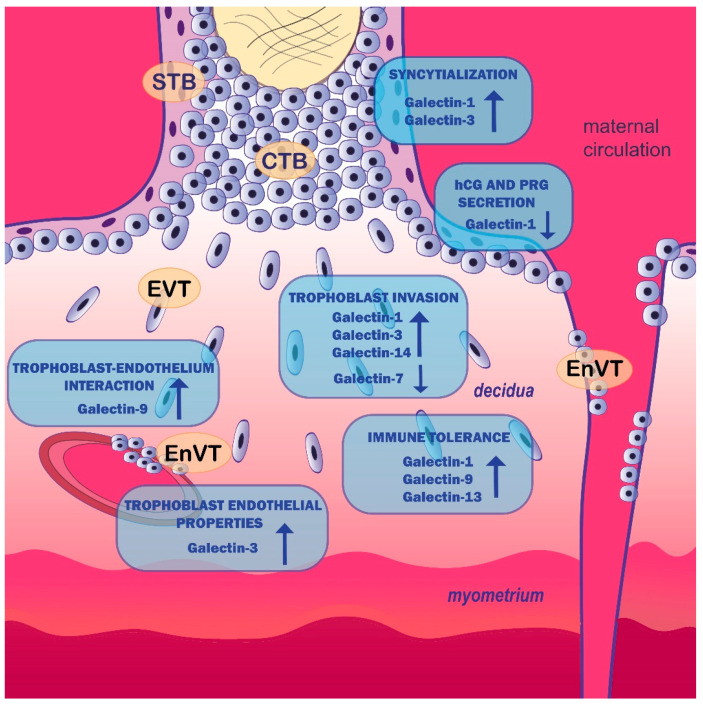
The proposed functions of galectins in the placentation process. STB—syncytiotrophoblast; CTB—cytotrophoblast; EVT—extravillous trophoblast; EnVT—endovascular trophoblast; PRG—progesterone; hCG—human chorionic gonadotropin. (Illustrated by Milica Jovanović Krivokuća).

Analysis of galectin signature in placenta tissue is of great importance for investigating their role in (pato)physiological processes related to pregnancy. So far, RNA and protein evidence have shown that galectins -1, -3, -7–-10 and -13–-17 are present in different trophoblast cell populations [8]. Three of them, galectin-13 (placental protein 13, -14 (placental protein-13-like) and -16) are exclusively expressed by the human placenta [6,9]. Moreover, the expression of some galectins, such as galectins -1, -3, -8, -13 and -14, is developmentally regulated and dependent on trophoblast differentiation. In the first trimester, placenta galectin-1 is abundantly expressed in syncytiotrophoblast (STB) and in EVT differentiating along the invasive pathway, but absent in the villous cytotrophoblast (VT) [10,11,12]. Placental anchoring villous and trophoblast subpopulations are presented schematically in Figure 1. Abundant expression of galectin-1 has also been described in the decidua of early gestation [12,13]. Jeschke et al. have reported the presence of galectin-2 in STB and EVT cells of third-trimester normal human placenta [14]. Interestingly, trophoblast galectin-2 expression was higher in male compared to female placentas [15]. Among trophoblast cell subpopulation, galectin-3 is detected in cytotrophoblast cells (CTB) of middle and distal cell columns, and in VT cells, as well [11,13]. This protein is also strongly expressed by decidual cells [16]. Presence of galectin-7 has been shown in STB and EVT and immune cells of first-trimester placental villi and decidua, as well as STB and endothelial cells of term placenta [16]. In addition to trophoblast, galectin-7 is expressed by decidua and endothelial cells of term placenta [9]. Galectin-8 was detected, like galectin-1 and -3, in STB, VT and EVT cells of first-trimester placenta [17]. Galectin-9 is mainly expressed in decidual cells and much less by CTB, while galectin-10 is found in first-trimester STB [18,19]. Galectins in the chromosome 19 gene cluster—galectin-13, -14, -16 and -17 are strongly expressed by STB cell of the placenta, but not by the underlying CTB [9,20]. Together with nuclear staining on STB, strong brush border membrane expression of galectin-13 is observed [21].

## 3. Modulation and Regulation of Galectin Expression in Pregnancy

The highly complex and versatile role of galectins during development and differentiation in both physiological and pathological pregnancy warrants finely tuned and coordinated modulation of their expression. However, the molecular mechanisms modulating galectin expression and activity are still largely underinvestigated.

Thus far, several factors implicated in the regulation of galectin expression were proposed: fetal sex, sex- and other hormones, redox status, pathogens and inflammatory mediators such as cytokines [22,23,24]. Sexually dimorphic expression of galectin-2, -13*/LGALS13* and -14/*LGALS14* in human placenta was shown under physiological conditions [15,24] and in intrauterine growth restriction (IUGR) [15], most likely reflecting genetic and hormonal differences between sexes. In addition, downregulation of galectin -4, -8 and -9 in male and overexpression of galectin-9 and -12 in female IUGR placentas compared to sex-matched placentas of fetuses with normal birth weight was also previously reported [25]. 

Besides genetic factors, epigenetic regulation of the expression of relevant genes could also be significant for the expression/function of the galectin family in pregnancy. Data on the role of DNA methylation in the control of expression of galectin genes are extremely sparse and the identification of coordinate mechanisms governing the activity of the different galectin genes is necessary for completing the complex puzzle of the galectin family. Among the early advances in this field is the recognition of estrogen-responsive elements (ERE) within the *LGALS1* gene, e.g., nuclear transcription factor-Y and activator protein-2, partially explaining the fluctuations of endometrial galectin-1 expression during the estrous or menstrual cycle and gestation concomitant with sex steroid hormone fluctuations [26,27,28]. Consistently, progesterone (PRG) and estrogen were shown to upregulate galectin-1 levels, whereas treatment with estrogen and progesterone receptor antagonists abolished this effect, suggesting the involvement of these nuclear receptors in *LGALS1* expression [28]. There is evidence that sex steroids regulate *LGALS1* expression in human endometrium during the menstrual cycle and also during decidualization in pregnancy [12]. Moreover, total cellular galectin-1 in HTR-8/SVneo cells was biphasically modulated by synthetic glucocorticoid dexamethasone, which was accompanied by a reduction of trophoblast cell invasion [29]. Secretion of galectin-1 was stimulated by PRG in HTR-8/SVneo cells [30]. Prolactin (PRL) stimulated the expression of intracellular galectin-1 in first trimester CTB and HTR-8/SVneo cells [31]. In vitro treatment of BeWo and RL95-2 cells as a model of trophoblast and endometrial cells, respectively, with 17β-estradiol (E2), PRG, and human chorionic gonadotropin (hCG) raised galectin-3 expression by these cells and also promoted its secretion from BeWo cells [32,33,34]. The same effect was shown upon stimulation of primary endometrial cells with E2, PRG [35] and hCG [34], indicating hormonal regulation of galectin-3 in trophoblast and endometrium and its involvement in endometrial receptivity.

Although it is generally taken that galectins and cytokines are reciprocally regulated under both physiological and pathological conditions, only the influence of galectins on cytokine production has been extensively researched [36]. Conversely, little is known about how cytokines affect galectin expression and function in a pro- or anti-inflammatory microenvironment, particularly in the context of pregnancy. The study of Ramhorst and colleagues [37] showed that exposure of JEG-3 choriocarcinoma cell line, commonly used as a model of human trophoblast, to recombinant human IL-2 and TNF-α led to substantial increase in the expression of galectin-1, suggesting a putative role for this galectin in the early stages of implantation when these cytokines play the leading role. On the other hand, considering the anti-inflammatory properties of galectin-1, its upregulation upon stimulation with proinflammatory cytokines may represent a compensatory mechanism to maintain homeostasis and prevent excessive inflammation and tissue damage [37].

It appears that conformational changes in galectin-1 mediated by redox status could be involved in the regulation of feto-maternal tolerance. Literature data indicate that fluctuations of redox status may regulate the diversity of biological functions of galectin-1 in physiological as well as in pathological processes. Thus, a high number of cysteine residues make galectin-1 highly sensitive to oxidation, which causes galectin-1 to lose lectin activity [38]. It has also been suggested that oxidized galectin-1 functions as a growth factor during axonal regeneration of peripheral nerves, while its reduced form appears to be critical for pro-apoptotic and immunoregulatory activity [38,39]. Although redox control of galectin-1 functions can be postulated for placentation and pregnancy as well, further functional experiments are needed to support this view. The effect of both oxidized as well as reduced galectin-1 is discussed in the following section. Moreover, as detailed further down in the text, endogenous factors such as ischemia/hypoxia and other non-infectious noxious stimuli may increase placental galectin release into the extracellular space upon stress, as part of the tissue alarmin system [40]. 

**Figure 2:**
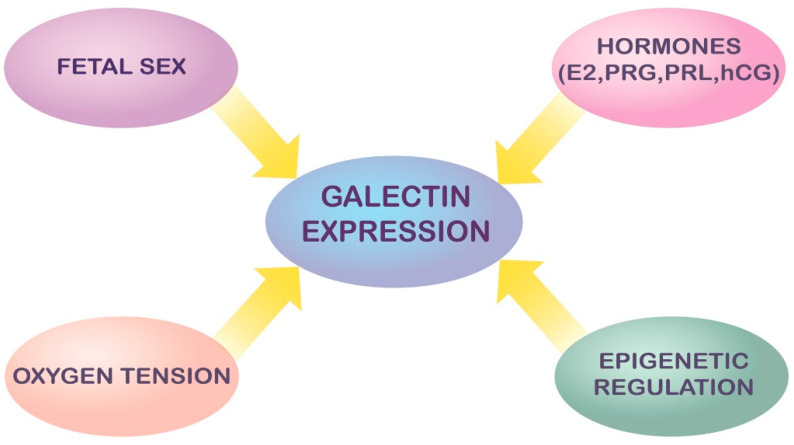
Suggested regulators of galectin expression in pregnancy.

Suggested modes of regulation of galectins in pregnancy are summarized schematically in Figure 2.

## 4. Galectins and Human Trophoblast Cell Function

A growing body of evidence suggests the involvement of galectin family members in placentation events beyond implantation, placental angiogenesis and in the establishment and maintenance of immune tolerance at the feto-maternal interface. The most studied galectins are galectin-1 and -3. Other galectin family members have also been implicated in some trophoblast cell functions. These findings are summarized in the following section.

The research from our lab has previously shown that galectin-1 may act as a part of trophoblast invasion machinery [41]. Both oxidized and reduced forms of recombinant human galectin-1 (rhgal-1) stimulated first-trimester CTB and HTR-8/SVneo cell invasion. This stimulation was reversed by neutralizing antibodies, as was by treatment with inhibitory sugar lactose, suggesting a lectin-type interaction-dependent mode of stimulation. These observations were replicated for the JAR trophoblast cell line, as well [42]. Proliferation of BeWo cells was inhibited by the treatment with galectin-1 [43]. Another study suggested that galectin-1 may play a key role in maternal immune regulation by modulating human leukocyte antigen G (HLA-G) expression on trophoblast cells [44]. Silencing of *LGALS1* and galectin-1 protein by siRNA downregulated the expression of HLA-G, a member of HLA class I heavy chain paralogues that is expressed by invasive EVT and recognized as essential for a mother’s immune tolerance towards the fetus [44]. Moreover, galectin-1 stimulates cell fusion of BeWo choriocarcinoma cells in a carbohydrate-dependent manner [45,46]. These cells are responsive to binding of galectin-1, showing decreased ability to produce hCG and PRG [47]. Several lines of evidence suggest that galectin-1 could influence EVT cell invasion through interaction with cell membrane glycoconjugates present on the β1 integrin subunit and MUC-1 mucin, or with ECM glycoproteins such as oncofetal fibronectin and laminin [47,48,49,50,51]. 

Together with galectin-1, galectin-3 also affects STB and EVT. Galectin-3 immunolocalization in EVT suggests its potential role in trophoblast invasion process. Recently, we investigated the possible involvement of galectin-3 in trophoblast invasion using in vitro functional assays [52]. Exogenously added recombinant human galectin-3 (rhgal-3) stimulated HTR-8/SVneo cell migration, which was reversed by a specific inhibitor of the galectin-3 carbohydrate binding domain I47 [53]. A similar effect was observed in a Matrigel invasion assay of both HTR-8/SVneo cells and isolated first-trimester CTB, when I47 was added. Attenuation of endogenous galectin-3 (by siRNA) led to a significant decrease in HTR-8/SVneo cell invasion, accompanied by decline in integrin β1 and matrix metalloproteinases (MMP)-2 and -9 [52]. Freitag and colleagues obtained similar results for HIPEC-65 cells, a CTB cell line, whose invasion was stimulated by rhgal-3, as well [54]. Moreover, exogenous galectin-3 enhanced the tube formation capacity of the EVT cell line SGHPL-4 [54]. In line with the proposed versatile role of galectin-3 in early pregnancy is the upregulation of this lectin in BeWo cells by hypoxia, a physiological condition characteristic for early placentation [55], and by treatment with E2, PRG and hCG [32]. Galectin-3 has been shown to stimulate BeWo cell fusion in vitro, suggesting its role in the syncytialization process [54].

Exogenous galectin-7 affected the interaction between trophoblast and endometrial cells, reducing cell–cell adhesion. When primary first-trimester trophoblast cells or HTR-8/SVneo cells were pre-treated with galectin-7, their capacity to adhere to endometrial epithelial cell monolayers was reduced, suggesting its role in embryo attachment and establishment of pregnancy [56]. Moreover, this galectin inhibited trophoblast outgrowth from first-trimester placental villous explants [57]. Galectin-9 has recently been shown to have immunomodulatory function at the feto-maternal interface, as it worked in cooperation with IL-27 to induce and promote differentiation of decidual T-cell immunoglobulin mucin domain-3 (Tim-3)+ CD4+ T cells into regulatory T cells [58]. Trophoblast cells secreted galectin-9, which induced the transformation of peripheral natural killer (NK) cells into a decidual NK-like phenotype, through interaction with Tim-3 [59]. Moreover, galectin-9 from HTR-8/SVneo cells protected the cells from NK cytotoxicity, acting as a proposed Tim-3 ligand [60]. These findings point to the possible role of trophoblast-derived galectin-9 in the maintenance of immune tolerance in early pregnancy. Apoptosis of HTR-8/SVneo cells was inhibited by galectin-9, as was proinflammatory cytokine production, while interaction with endothelium was increased [61] in a c-Jun N-terminal kinase (JNK)-dependent manner, adding yet another possible role of this lectin for pregnancy success. Galectin-13 is another galectin with proposed immunomodulatory functions, as this placenta-derived lectin induced apoptosis of activated T cells in vitro, and diverted and killed T cells and macrophages in the maternal decidua, polarized neutrophils towards permissive phenotype for placental growth [62,63]. Galectin-14 promoted trophoblast cell migration and invasion by stimulating the expression of MMP-9 and N-cadherin through Akt phosphorylation, while knockdown of galectin-14 in primary trophoblast had the opposite effect. On the other side, overexpression of galectin-14 in HTR-8/SVneo cells promoted cell migration and invasion and upregulated the abovementioned molecules [64]. Recent research suggests that galectin-13 and -14 at the feto-maternal interface have immunoregulatory and vascular effects, as was found for galectin-1 and -3 [6]. However, to better understand their role at the feto-maternal interface, more experiments are of great importance.

Taken together, galectins act to modulate trophoblast cell functions and immune tolerance at the feto-maternal interface. The proposed involvement of galectins in the placentation process is presented in Figure 1 and summarized in Table 1. Since galectin family members bind to various glycoconjugates and have pleiotropic roles, we assume that there is a whole spectrum of galectin functions in early pregnancy events yet to be elucidated.

## 5. Galectins in Pregnancy-Related Disorders

There is an increasing number of investigations describing changed or dysregulated galectin expression in different pregnancy pathologies. Some of the complications are described during placenta development, while other are linked with third-trimester pregnancy. Here, we focus on the galectin family members in pregnancy loss, gestational diabetes mellitus (GDM), IUGR and pre-eclampsia (PE). Implication of galectins in pregnancy-related disorders in summarized in Table 1.

### 5.1. Galectins in Pregnancy Loss

The majority of adverse pregnancy outcomes that result in pregnancy loss can trace their origin to early defects in pregnancy establishment. It is known that miscarriage, stillbirth and recurrent pregnancy loss (RPL) can stem from multiple causes that implicate increased uterine and placental dysfunction, environmental exposures, advanced maternal age and presence of maternal comorbidities (systemic inflammatory diseases, endocrine diseases, etc.), excluding causes driven by chromosome errors (aneuploidy) in the conceptus [111]. Although underlying causes for pregnancy loss differ between women, experts agree that certain pathological mechanisms probably converge to the common pathway to eventually trigger pregnancy loss. Much progress has been made in the past decade in understanding the molecular pathways that promote placental insufficiency and the subsequent progression to pregnancy loss. Important molecular mediators that take critical roles at different developmental stages are currently being identified and explored [112]. So far, very few studies recognize galectin family members as factors related to pregnancy loss. In STB, expression of galectin-1 was significantly lower after miscarriage and RPL [65,66]. In contrast, decidual galectin-1 was upregulated in RPL [113]. The same study showed that galectin-2 was downregulated in VT and EVT after spontaneous abortion (SA) and RPL, as well as galectin-7 and -10, but only in VT [66]. On the other hand, the expression of galectin-3 was not changed in SA and RPL. However, Gao and Fang showed that galectin-3 was markedly decreased in serum, decidua and the placental villi in the group of women with missed abortions (MA) [69]. The association between abnormal expression of galectin-3 and excessive apoptosis in placental villi was also shown in cases of SA as well as MA [68]. These authors reported that there are different patterns of galectin-3 expression in SA and MA before and after the fourth week of pregnancy. They speculated that early downregulation of galectin-3 prior to the fourth week of gestation in MA cases causes a compensatory mechanism in trophoblast cells that start to secrete excessive galectin-3 in response. After the fourth week, excessive galectin-3 secreted by VT cells leads to massive apoptosis of endometrial cells, which affects the normal development of villi in early pregnancy, and potentially leads to MA [66]. Recent study suggests that if controlled trophoblast invasion and apoptosis and the “inhibition–expression” balance of galectin-3 is broken, a pathological pregnancy may occur, and the success of pregnancy may be compromised [68]. Furthermore, it was suggested that imbalance between the extracellular and intracellular galectin-3 levels can influence cell apoptosis in placental villi, leading to defects in early placental development and ultimately resulting in pregnancy loss. Although association studies are uncertain in linking galectin family members to pregnancy loss, these lectins as molecules associated with placental development may be involved in mechanisms underlying this pathology [67]. 

### 5.2. Galectins and GDM

GDM is a condition where there is an abnormal blood glucose level in pregnancy, but without previous diagnosis of diabetes [114]. GDM develops in about 3–5% of pregnancies [115]. Diabetic insult at the beginning of gestation may have long-term effects on placental development [116]. If the duration or level of the diabetic insult surpasses the placental capacity to mount adequate responses, then excessive fetal growth may occur [116]. Other perinatal risks also include shoulder dystocia, birth injuries and hypoglycemia [117]. Over the past decade, data regarding altered expression of some galectin family members in placentas of GDM patients suggest their involvement in GDM pathophysiology.

In normal pregnancy, serum galectin-1 levels are increased during gestation [44], whereas in GDM, there seems to be an unchanged galectin-1 secretion pattern [70]. One study demonstrated (*ex vivo*) that changes in glucose concentrations at the feto-maternal interface decrease galectin-1 secretion by the placenta [70]. These results agreed in GDM patients, where there was an inverse association between glucose and galectin-1 [70]. Additionally, it was found that there was an association between the galectin-1 5′ regulatory *LGALS1* SNP rs4820294 (C/T) gene polymorphism and GDM-complicated pregnancy [70]. However, a larger sample size is needed to confirm this association [70].

A very recent study showed increased galectin-2 expression in STB as well as in decidua of GDM placentas. This study leads to two possible conclusions about the role of galectin-2 dysregulations in the pathophysiology of GDM [71]—whether the increased galectin-2 expression is a reaction to the inflammatory state of GDM or if it contributes to its development [71]. Therefore, further research is needed to clarify its role in GDM and possible therapeutic implications.

Galectin-3, especially its circulating levels, has also been described in GDM. It was found that women in the first trimester had higher levels of galectin-3 and were more likely to develop GDM later in the pregnancy than women found to have low levels of galectin-3 [72], suggesting potential for galectin-3 to be used as a novel early biomarker for the development of GDM [72]. However, larger studies are needed to improve risk stratification models [72].

It has been shown that circulating levels of galectin-3 increased in maternal circulation with progression of normal pregnancy during the second and third trimesters [54]. Furthermore, it was shown that galectin-3 was mostly expressed in endovascular extravillous cytotrophoblast (EnVT) during the first trimester, where it stimulates important trophoblast functions, such as invasion and tube formation, which affect healthy placental development [54]. It was proposed that trophoblast cells could be one of the sources of the circulating lectin because the increase in galectin-3 in maternal peripheral levels corresponds with the period of placental growth during the second and third trimesters [54]. The same study reported that patients who developed GDM had reduced levels of serum galectin-3, which was only evident during the third trimester [54]. This could indicate that galectin-3 is sensitive to the hormonal and metabolic changes that characterize GDM [54]. An additional study also demonstrated that circulating galectin-3 levels are higher in subjects with GDM and also correspond to increased risk of GDM [73]. Moreover, galectin-3 and PRG levels were highly correlated, as well as insulin resistance [73]. The study showed that the association between galectin-3 and GDM is not mediated by adiposity because elevated galectin-3 levels were related to the increased risk of GDM when also adjusting for current body mass index (BMI) [73]. The study showed no independent correlation between circulating galectin-3 levels and current BMI [73]. It was suggested that there is a possible contribution of galectin-3 to GDM that mostly involved pathways in insulin resistance and not insulin secretion [73]. There is an underlying association between galectin-3 and insulin resistance in that galectin-3 directly binds the insulin receptor, which inhibits downstream insulin receptor signaling [118]. It was speculated that this may be associated with galectin-3 and insulin resistance in GDM, but further research is required to investigate this observation [73].

In another study, galectin-3 mRNA and protein expression was found to be increased in GDM maternal blood samples and placental tissue, and decreased in cord blood [74]; however, a different study found that when mothers presented with GDMs, cord blood galectin-3 was significantly increased [75].

Studies linking galectin-13 and GDM have shown dysregulated expression of this protein in placental tissue as well as changes in its serum concentrations. Lack of galectin-13 and, therefore, lack of its anti-inflammatory functions may play a part in the inflammation process of the placenta [76]. This inflammatory process could then be imbalanced, which may lead to GDM [76]. As mentioned, galectin-13 was found, specifically in STB and EVT [76]. However, galectin-13 expression was markedly lower in the above-named parts of GDM placentas [76]. In addition, galectin-13 serum levels were much lower in GDM pregnancies than in normal term pregnancies [76]. Another study found that women with GDM in the early second trimester had significantly higher galectin-13 levels in the serum [77], suggesting that this protein may be of importance in the prediction of subsequent GDM, and this was consistent with previous studies. However, in a different study, it was found that macrosomia at birth in pregestational type-1 and type-2 diabetes (PGDM) pregnancies may be predicted by normal levels of galectin-13 together with plasma protein A (PAPP-A), A disintegrin and metalloproteinase 12 (ADAM12) and placental growth factor (PlGF) in the first trimester of pregnancy [119]. 

Taken together, these findings suggest that galectin-3 and galectin-13 measurements in GDM might be of value, but additional studies are needed to confirm their potential use as biomarkers. 

### 5.3. Galectins in Inflammation/Infection in Pregnancy

As molecules ubiquitously expressed in mammalian immune cells and tissues galectins are engaged in both innate and adaptive immune responses. Acting extra- and intracellularly, due to their cross-linking activity and multifaceted biochemical and biophysical properties, galectins affect leukocyte adhesion, migration, differentiation, proliferation and apoptosis; production of cytokines and other mediators; chemoattraction; receptor function; etc. [36]. Like cytokines, galectins may exhibit both pro- and anti-inflammatory features depending on the cellular/tissue context, contributing to the activation and/or resolution of the inflammatory/immune response [120,121]. 

Galectins are considered indispensable for the immune-endocrine crosstalk at the feto-maternal interface and the establishment and maintenance of pregnancy and feto-maternal immune tolerance [8,122]. Although infection and non-infectious inflammatory/immune insults in pregnancy are well-defined risk factors for various obstetric complications [123,124,125,126], the role of the galectin family in the immune pathways underlying these complications remains a major knowledge gap. 

Irrespective of the etiology, an acute or chronic inflammatory process could cause a breakdown of the tolerogenic mechanisms at the feto-maternal interface and lead to immune deregulation with possibly deleterious health outcomes for both the mother and the fetus [126]. One of the possible targets of the inflammation-associated immune dysregulation at the feto-maternal interface could be the Tim-3/galectin-9 axis, which was suggested to regulate immune cells to maintain feto-maternal tolerance [83]. Consistently, data from animal models indicate that impairment of the Tim-3/galectin-9 pathway in pregnancy contributes to the failure of immunotolerance. 

Another link between systemic infection and placental galectin dysregulation was seen in an animal model of periodontitis, a common gestational infection caused mainly by Gram-negative pathogens, which was shown to be associated with a 7.9-fold increased risk of preterm birth (PTB)/low birth weight in humans [80]. Research on a murine model of dental infection with *Porphyromonas gingivalis* (P.g.) has shown feto-placental barrier invasion by this pathogen and development of placental inflammation and PTB through mechanisms involving upregulated galectin-3 expression. 

Although maternal systemic infections are often associated with PTB and other adverse pregnancy outcomes, intrauterine infections are considered to be a pivotal risk factor for obstetrical complications [126]. Due to their high-affinity β-galactoside-binding properties, some galectins are able to detect microorganisms by binding to their glycans acting as receptors for pathogen-associated molecular patterns (PAMPs), i.e. pattern recognition receptors (PRRs) [40,127]. Such recognition may initiate immune responses leading to clearance of microorganism or, conversely, it may facilitate establishment of infection [40]. Results from our laboratory suggest a role for galectin-3 in the inflammatory response in chorioamniotic infection and preterm premature rupture of membranes (PPROM), considering its overexpression in chorioamniotic membranes in women with PPROM and histologically confirmed intraamniotic infection compared to those with PPROM without chorioamnionitis [79]. The biological implications of the upregulated galectin-3 expression in chorioamnionitis remain to be elucidated. Significantly increased expression of the immunoregulatory galectin-1 in the chorioamniotic membranes was also shown in cases of PPROM with chorioamnionitis compared to PPROM cases without chorioamnionitis. Its spatial and temporal expression also changed with the evolution of the disease. The overabundant expression of galectin-1 was linked with inflammation and tissue remodeling, leading to weakening of the membranes and contributing to their rupture [78]. On the other hand, considering its immunoregulatory properties, the overexpression of galectin-1 by the human chorioamniotic membrane in chorioamnionitis could represent a local protective response to counteract inflammation and establish immunological tolerance [78]. Similarly, increased galectin-1 expression in the placenta as a possible response to maternal exaggerated systemic inflammation was also seen in severe PE [128]. Given the increased vesicular release of galectins upon cellular stress and damage or traumatic cell death, a role for some galectins as classic stress sensors, i.e., damage-associated molecular patterns (DAMPs) or “alarmins”, was also suggested. Released DAMPs signal tissue damage and trigger effector responses in immune cells, thereby orchestrating the inflammatory/immune response in parallel with the PAMP system [40]. In line with this notion are data indicating that danger signals (either of host or pathogen origin) released at the feto-maternal interface and recognized by trophoblasts expressing Toll-like receptors (TLRs), classical PRRs activated by PAMPs and DAMPs, may induce an inflammatory response that creates an aggressive cytokine microenvironment leading to the development of PE [129,130]. Excessive release through extracellular vesicles of the placental protein 13 (PP13/galectin-13), a member of the galectin family exclusively expressed in anthropoid placental tissue, primarily by STB [21], was shown in preterm PE and Hemolysis, Elevated Liver enzymes and Low Platelets (HELLP) syndrome, leading to increased PP13 concentrations in maternal circulation at the clinical onset of the syndromes [131]. Increased expression of PP13 was also observed when BeWo cells were put under conditions mimicking ischemic stress [131], which is a major pathophysiologic mechanism of PE [132], suggesting a role for PP13 as a placental “alarmin” to the immune system, whether in PE, ischemia or other stress conditions that pose danger to the organism [62,131]. 

Finally, although anti-galectin autoantibodies (aAbs) can be detected in sera from healthy people, elevated levels of circulating anti-galectin aAbs may be seen in infectious and autoimmune diseases. Increased titers of anti-galectin-1 aAbs were reported in autoimmune and infectious neurological disorders [86] and uveitis [87], Chagas disease [133] and systemic lupus erythematosus (SLE) [88]. High titers of anti-galectin-3 aAb were seen in cutaneous vasculitis and nephritis in SLE [90,91], and especially in polymyositis/dermatomyositis [92]. Anti-galectin-4 and -8 aAbs were found in SLE and rheumatoid arthritis (RA) [89,93]; anti-galectin-7 aAb in SLE; and anti-galectin-8 and -9 aAbs in SLE and RA [89]. The implications of the increased titers of anti-galectin aAbs for pregnancy outcomes are not fully elucidated. However, the study by Sarter and her collaborators revealed that the occurrence of anti-galectin aAbs in SLE and RA was highly associated with secondary anti-phospholipid syndrome (APS), clinically characterized by recurrent miscarriages and other thrombotic events [89]. In this study, anti-galectin-2 aAb levels in SLE patients particularly highly correlated with the appearance of the obstetric and thrombotic complications of APS, suggesting that this parameter may also serve as an additional biomarker for APS [89].

### 5.4. Galectins in PE

PE is a pregnancy-specific, multisystem disorder, related to elevated morbidity and mortality risk for both the mother and the newborn. With complex and still not fully explained pathophysiology, PE is primarily manifested by hypertension and liver and kidney failure [134]. Depending on the time of onset, PE is marked as early-onset (before the 34th week of gestation) or late-onset PE (after the 34th week of gestation) [135]. The main pathological feature of PE is impaired placentation due to inadequate trophoblast invasion; incomplete transformation of uterine spiral arteries, causing impaired placental perfusion; and enhanced maternal systemic inflammatory response to factors released by stressed STB into maternal circulation [134]. Of the molecules with deregulated expression in PE, some galectin family members are recognized as promising candidates for better understanding of PE pathology. 

Histochemical analysis of placental tissue showed overexpression of galectin-1 in PE placentas compared to placentas in normal pregnancy, with particularly intensive staining of EVT and decidual cells [94]. Than and co-workers reported similar findings, reporting increased expression of galectin-1 on both mRNA and protein levels in placental tissue of women who developed severe PE [78]. As aforementioned, the overexpression of galectin-1 in PE might be induced by a systemic inflammatory reaction in women with PE as a local reaction that should silence the maternal immune response. Namely, pregnancy represents an immune challenge for the maternal organism and galectin-1 plays an important role in the tight regulation of the immune tolerance towards the fetus. In women who developed PE, T and NK cells expressed lower galectin-1 positivity than in women with non-complicated pregnancy [96]. On the other hand, downregulation of galectin-1 was also seen in placentas with early-onset PE [97].

Inadequate trophoblast invasion and remodeling of uterine spiral arteries during early stages of placenta development leads to shallow implantation that subsequently might be related to PE occurrence. In that context, interaction of galectin-1 with α1 and β1 integrins seems to be related to PE [136]. This could be the consequence of changed galectin-1 expression and modified integrin glycosylation [137]. Similar findings were observed in placenta from HIV-negative and HIV-positive women, where expression of galectin-1 was significantly decreased in pre-eclamptic compared to normotensive patients, irrespective of HIV infection status [95]. These conflicting data regarding overexpression or downregulation of galectin-1 should not be surprising, given that PE is not a single pathological entity, but rather a complex syndrome with different etiology and pathogenetic mechanisms. 

Besides evaluation in placental tissue, galectin-1 was also measured in maternal circulation and studied as a potential biomarker in PE. It was found that galectin-1 levels in maternal circulation increase during non-complicated pregnancy progression [44]. In addition, in women who developed PE, circulating galectin-1 levels were higher compared to normotensive pregnancy of the same gestational age. While serum galectin-1 levels were higher in preeclamptic women, decreased levels of galectin-1 in maternal circulation between the 18th and 24th gestational week were a prognostic indicator of subsequent PE development [98]. These findings suggest that measurement of galectin-1 in maternal blood could be used for noninvasive testing as a predictive marker of PE risk. 

Recently, galectin-2 was identified as one of the molecules with decreased expression in pre-eclamptic EVT at mRNA and protein level, regardless of the time of PE onset [99]. Consistent with this, Charkiewitz and co-workers found significantly lower galectin-2 levels in peripheral blood of women with PE [138]. So far, it is not clear whether decreased galectin-2 expression is liable for PE development or whether it is a consequence of shallow trophoblast invasion in the first trimester.

Several studies showed galectin-3 overexpression in PE patients. Increased galectin-3 mRNA and protein levels were detected in PE placental tissue in comparison to normal pregnancy placentas [100]. Although different cell types of term placenta express galectin-3, increased expression of this lectin in EVT and STB was connected to PE [94,100,101]. Furthermore, serum galectin-3 levels were significantly higher in PE patients compared to those with uncomplicated pregnancy [101,102]. Pankiewicz and colleagues found a significant correlation between galectin-3 serum levels and its expression in STB in PE patients, suggesting that increased secretion of galectin-3 from the placenta could be at least partially contributing to elevated galectin-3 in the circulation [101]. In the same work, galectin-3 expression in EVT correlated with serum levels of soluble FMS-like tyrosine kinase-1 (sFlt-1), one of the hallmarks of PE [101]. On the other hand, Nikolov and associates did not detect differences in serum galectin-3 levels between patients with pre-eclamptic and normotensive pregnancies [139]. This discrepancy could be at least partly due to differences in study protocols and methods for measuring galectin-3 serum levels. Nevertheless, available data implicate galectin-3 in PE development and further research is needed to determine its role in the pathophysiology of this pregnancy disorder and the potential use of galectin-3 as a biomarker for PE.

Galectin-7 is one of the newly identified potential prospective serum biomarkers for PE [16]. Menkhorst and colleagues demonstrated elevated galectin-7 serum concentrations in the 10–12th and 17–20th week of gestation in women who developed PE in comparison to healthy pregnancies [16]. Although a larger sample number is needed to establish galectin-7 as a predictive PE biomarker, it would be of great importance to combine galectin-7 serum levels with other proteins altered in PE, since so far, there is no available predictive biomarker(s) for this pregnancy-related pathology. 

Galectin-9 is another family member with a possible role in PE pathogenesis [84]. Its altered expression, together with cell surface receptor Tim-3, is detected on peripheral blood lymphocytes in early-onset pre-eclamptic women and could result in an increased inflammatory response in PE. However, so far, it is not clear whether Tim-3/galectin-9 interactions display different immunological responses in PE compared to healthy pregnancy. 

To date, the most studied galectin family member in the PE context is galectin-13 because of its potential as a useful biomarker for this pregnancy disorder. Its decreased expression at mRNA and protein levels was described in both early- and late-onset PE in comparison to healthy controls [103,104]. Of great importance is the study that showed reduced mRNA galectin-13 expression from chorionic villi samples at the 11th week of gestation in women who subsequently developed PE, which could be the earliest PE pathological indication [140]. Interestingly, while downregulation of galectin-13 was described in STB of PE placentas, STB microvillous membranes expressed increased galectin-13 level in PE compared to healthy controls [103]. As a consequence, concentrations of circulating galectin-13 are elevated in PE during the third trimester. Evidence also supports galectin-13 subcellular redistribution to the juxta-membrane region of the STB in PE in comparison to healthy controls [131]. As discussed above, galectin-13 may have various functions, especially in early pregnancy, and its importance for placentation may be reflected by the observed decreased placental expression and maternal serum concentrations in the first trimester in PE. The only source of galectin-13 at mRNA and protein level in maternal circulation is the placenta, which makes this molecule a suitable candidate as biomarker related to pregnancy pathologies, including PE. Although low galectin-13 mRNA in maternal circulation indicated that alterations of its expression connected to PE appear very early in pregnancy, the predictive value of galectin-13 mRNA is currently limited because of the low amounts of trophoblast mRNA [140,141]. At the protein level, after the detected low levels in the first trimester, galectin-13 serum concentration rapidly increases in PE compared to healthy pregnancy starting from the second trimester. In search of a sensitive and reliable predictive PE biomarker, galectin-13 was evaluated by a meta-analysis of data based on studies that used immunoassay platforms [142]. This review confirmed lower circulating galectin-13 levels in women who subsequently developed PE and showed that its combination with other biochemical markers, Doppler pulsatility index and mean arterial pressure, increase galectin-13’s predictive value as a PE biomarker [143,144,145]. So far, measurement of galectin-13 levels, Doppler pulsatility index and pulse wave analysis is a promising predictive combination of PE in women with a priori high risk for the condition [105]. These results and use of a wide biomarker panel could represent a viable strategy for PE prevention and management. 

### 5.5. Galectins and IUGR 

IUGR is defined as the pathologic inhibition of intrauterine fetal growth and the failure of the fetus to achieve its growth potential [146]. It has been associated with different etiologies encompassing fetal (genetic abnormalities), maternal (vascular diseases, persistent hypoxia, poor nutrition, smoking, alcohol consumption, etc.) and placental factors [147]. IUGR is an important public health problem worldwide. It is well known that perinatal mortality and morbidity is markedly increased in IUGR fetuses [148]. The prevalence is about 8% in the general population [146]. It has been shown that 52% of stillbirths are associated with IUGR and that 10% of perinatal mortality is a consequence of IUGR. Besides fetal distress, stillbirth and other adverse perinatal outcomes, IUGR is correlated with long-term consequences in adult age, such as cardiovascular disease and metabolic syndrome [149]. Bearing in mind that adequate fetal growth is essential for later development and health, and that there is a lack of markers for early detection and appropriate management, in-depth research into this pregnancy complication and predictive factors is necessary [150]. 

Although several analytes/metabolites have been studied with the aim of finding a relevant marker linked to abnormal fetal growth screening, none are generally applied in clinical practice as a single predictive marker [151]. Summarizing knowledge about IUGR pathology and the role of galectins in PE, which are closely linked to the development of IUGR, it appears conclusive to look for galectin expression in cases of IUGR as the first step in identifying possible correlations [15]. 

Various galectin dysregulations are found in this pregnancy complication [15]. The expression of galectins in placenta appears to be downregulated in cases of IUGR. Galectin-2 and galectin-13 seem to be highly correlated in their placental expression in all placental compartments. The expression scheme of galectin-3, the only chimera-type galectin, seems to be independent of the prototype galectins-1, -2 and -13 [15].

Pregnant women with single birth IUGR with or without comorbidities (PE, GDM) and pregnant women with normal singleton pregnancy without complications or comorbidities were enrolled in a recent study [106] in which galectin-1 expression in the serum and placenta was investigated. The obtained results revealed that the galectin-1 serum level significantly decreased in the IUGR group compared with the control. Furthermore, the serum galectin-1 level positively correlated with birth weight. In the placenta, the galectin-1 expression level also decreased significantly in the IUGR group compared with the control group [1]. These results suggest that galectin-1 exhibits low expression in the serum and placenta of pregnant women with IUGR and that this lectin may be involved in the pathogenesis of IUGR and could represent a new diagnostic marker of this disease. 

It was found earlier that the difference in galectin-1 expression in the placenta between the IUGR group and the control group was not statistically significant; there was no change in expression in female placentas compared to controls. However, expression of galectin-2 in male IUGR placentas showed a six-fold decrease compared to controls [15]. These studies may serve to caution investigators to factor fetus sex as an important variable when designing, analyzing and reporting research on possible changes in galectin expression/function in normal and pathological pregnancies.

It is worth noting that galectin-3 also plays a role in human IUGR pathology. Placental galectin-3 expression is downregulated in human pregnancies complicated with IUGR. Namely, Freitag and colleagues showed that galectin-3 within the maternal compartment is required for proper placental development and fetal growth [67]. Their findings identify galectin-3 as a key component of the molecular program of decidual/placental development and offspring health, as well as a potential target for future strategies aimed at minimizing adverse outcomes in pregnancies at high risk of IUGR.

In experimental animals, galectin-3 loss of function during gestation altered the decidual compartment, favoring a pro-inflammatory milieu. In the placental compartment, lack of galectin-3 compromised placental vascularization and perfusion, resulting in placental insufficiency (reduced placental weight, reduced trophoblast layers, increased pro-inflammatory cytokines expression, reduced placental labyrinth total vessel length and vessel area) and the subsequent development of asymmetric IUGR in mice (as denoted by an increased brain-to-liver weight ratio). The reduced fetal weight in galectin-3 deficient fetuses was accompanied by a delay in fetal development [67]. 

It was also shown that galectin-3 expression was associated with being small for gestational age, given that cord blood of infants with a birth weight below the 10th percentile for their gestational age had higher galectin-3 levels than appropriate-for-gestational-age infants. Considering the proinflammatory role of galectin-3, the higher expression of this lectin in small-for-gestational-age infants might be a reflection of inflammation due to chronic hypoxia of the fetus [107].

The study by Boutsikou et al. was conducted with the aim to determine levels of galectins-1 and -3 in IUGR, large- (LGA) and appropriate-for-gestational-age (AGA) pregnancies [75]. The authors concluded that IUGR, LGA and AGA groups did not differ in galectin-1 and -3 concentrations in umbilical cord blood and that the lower galectin-1 levels in older mothers, and increased galectin-3 levels in GDM, possibly reflect their angiogenic activity. However, in EVT, the expression of galectin-1 and galectin-3 was unchanged in IUGR placentas compared with normal controls [94]. 

Altered expression of galectin-13 is also described in PE and early fetal growth restriction [143,152]. According to the study conducted by Burger and co-workers, a bi-modal effect of an abnormal PP13 level in placental insufficiencies was found [108]. Namely, in the first trimester, PP13 is present in lower than normal levels in the maternal blood serum of women suffering from PE and IUGR. At the second and third trimesters, PP13 was shown to be significantly higher in PE, IUGR and pre-term delivery compared to normal serum samples. While Chaftez et al. showed that low levels of first-trimester PP13 were associated with preterm birth in women with IUGR [109], Cowans and colleagues found that decreased serum levels of galectin-13 were not significantly correlated with the studied adverse pregnancy outcomes of IUGR, preterm low birth weight and intrauterine fetal demise [110]. As for placenta tissue, galectin-13 expression was strongly decreased in VT and EVT in IUGR-complicated pregnancies of male fetal gender [15].

Knowing that delivery of a sub-optimally grown and often preterm baby, with a guarded short-term and long-term prognosis, is the end result of IUGR, identification of the mechanisms behind this disorder is of major scientific and therapeutic interest [153]. Further studies are required to evaluate if galectin measurement has any value in the early assessment of pregnancies. 

## 6. Conclusions

The data presented here were selected with the aim of contributing to our understanding of the roles of galectin family members in processes leading to the establishment and maintenance of pregnancy. This has been particularly well described for trophoblast cell function, while data regarding clinical relevance of galectins have only just started to accumulate. Since their altered expression is linked to different pregnancy-related pathologies, much remains to be learned about the possibility that galectins can be suitable diagnostic and predictive biomarkers, but also exploited as therapeutic tools. 

## Figures and Tables

**Table 1:** Involvement of galectins in pregnancy-related processes and pathologies.

Process/Pathology	Involvement of Galectins	Reference
**Trophoblast cell function/placentation**	**Galectin-1** stimulates first-trimester cytotrophoblast, HTR-8/SVneo and JAR cell invasion	[41,42]
	**Galectin-1** decreases BeWo cell proliferation	[43]
	**Galectin-1** modulates HLA-G expression on trophoblast cells, suggesting its role in immune tolerance	[44]
	**Galectin-1** stimulates syncytialization of BeWo cells but decreases human chorionic gonadotropin and progesterone production	[45,47]
	**Galectin-3** stimulates HTR-8/SVneo cell migration and HTR-8/SVneo cell, first-trimester CTB and HIPEC-65 cell invasion	[52,54]
	**Galectin-3** increases endothelial-like properties of EVT cell line SGHPL-4	[54]
	**Galectin-3** stimulates syncytialization of BeWo cells	[54]
	**Galectin-7** decreases first-trimester trophoblast cells’ and HTR-8/SVneo cells’ capacity to adhere to endometrial epithelial cells and first-trimester EVT outgrowth from placental explants	[16,57]
	**Galectin-9** derived from trophoblast cells promotes immune tolerance at the feto-maternal interface	[58,59,60]
	**Galectin-9** inhibits the apoptosis and proinflammatory cytokine production of HTR-8/SVneo cells and increases the interaction with endothelium in a JNK-dependent manner	[61]
	**Galectin-13** induces apoptosis of activated T cells in vitro, diverts and kills T cells and macrophages in the maternal decidua and polarizes neutrophils towards permissive phenotype for placental growth	[62,63]
	**Galectin-13** may induce angiogenesis at the feto-maternal interface	[6]
	**Galectin-14** promotes trophoblast cell migration and invasion by stimulating the expression of MMP-9 and N-cadherin through Akt phosphorylation	[64]
	**Galectin-14** may function in angiogenesis	[6]
**Pregnancy loss**	**Galectin-1** is significantly lower in placenta tissue after miscarriage and RPL	[65,66]
	**Galectin-2** is downregulated in VT and EVT after SA and RPL	[66]
	Maternal deficiency of **galectin-3** is associated with structural alterations in placenta, with reduced trophoblast layers and a corresponding enlarged maternal decidua. The absence of galectin-3 also results in reduced total vessel length and vessel area, suggesting placental malperfusion.	[67]
	Excessive **galectin-3** after 4th week secreted by VT leads to massive apoptosis of endometrial cells, which affects the normal development of villi in early pregnancy, and potentially leads to missed abortion.Imbalance between extracellular and intracellular **galectin-3** levels can influence cell apoptosis in placental villi, leading to defects in early placental development and ultimately result in pregnancy loss.	[68]
	**Galectin-3** is markedly decreased in serum, decidua and the villi in the group of women with missed abortions	[69]
	Expression of **galectins-7** and **-10** is decreased in VT after SA	[66]
**Gestational diabetes mellitus (GDM)**	Serum galectin-1 levels are increased during gestation, whereas in GDM, its secretion pattern seems to be unchanged	[44,70]
	In GDM patients, there is an inverse association between glucose and **galectin-1** compared to normal pregnancies	[70]
	Possible relation of **galectin-2** overexpression to pathophysiology of GDM	[71]
	Women in the first trimester had higher levels of **galectin-3** and were more likely to develop GDM later in the pregnancy than women found to have low levels of galectin-3	[72]
	Circulating **galectin-3** levels are higher in subjects with GDM and also correspond to increased risk of GDM	[73]
	**Galectin-3** mRNA and protein expression are increased in GDM maternal blood samples and placental tissue, and decreased in cord blood	[74]
	Cord blood **galectin-3** is significantly increased in pregnancies with GDM	[75]
	**Galectin-13** expression is markedly lower in the placenta of GDM pregnancies. Galectin-13 maternal serum levels at term are significantly lower, while in the early second trimester, significantly lower than in normal pregnancies.	[76,77]
**Inflammation** **/infection**	**Galectin-1** expression increases in chorioamniotic membranes, promoting weakening of the membranes and contributing to their rupture, or as a compensatory response to counteract inflammation and retain immunological tolerance	[78]
	**Galectin-3** is overexpressed in chorioamniotic membranes in women with PPROM, suggesting its role in the pathogenesis	[79]
	**Galectin-3** expression increases in placenta and amniotic fluid upon *Porphyromonas gingivalis* placental invasion and development of inflammation, further potentiating local cytokine production and activation of myometrium	[80,81]
	**Galectin 3** inhibits CD66a expression by intermediate trophoblast and endometrial epithelium/endothelium, thus contributing to placental abruption and preterm birth	
	Tim-3/galectin-9 axis impairment contributes to failure of immunotolerance by a shift towards the proinflammatory M1 phenotype of decidual macrophages, increased placental expression of TNF-α, IL-1β and iNOS, and reduced expression of TGF-β, IL-10 and Arginase-1, accompanied by inadequate trophoblast invasion, impaired spiral artery remodeling and fetal capillary development	[59,82,83,84,85]
	**Anti-galectin-1 Abs** are found in increased titers in autoimmune uveitis, and SLE	[86,87,88]
	**Anti-galectin-2 Abs** in SLE (highly associated with secondary anti-phospholipid syndrome)	[89]
	**Anti-galectin-3 Abs** in SLE, polymyositis/dermatomyositis	[90,91,92]
	**Anti-galectin-4** in SLE and RA	[89]
	**Anti-galectin-7 Abs** in SLE	[89]
	**Anti-galectin-8** and **-9** in SLE and RA	[89,93]
**Pre-eclampsia** **(PE)**	**Galectin-1** overexpression in PE placentas, compared to placentas in normal pregnancy	[78,94]
	Downregulation of **galectin-1** in early-onset PE placentas	[95]
	**Galectin-1**-expressing peripheral blood T and NK cells proportion is decreased in women who developed PE compared to normal pregnancy	[96]
	Downregulation of **galectin-1** in early-onset PE placentas	[95,97]
	Low serum **galectin-1** levels during the second trimester might be a PE risk	[97,98]
	**Galectin-2** is downregulated at protein and mRNA levels in EVTs in PE placentas **Galectin-2** serum levels are lower in PE patients compared to patients with uncomplicated pregnancies	[99]
	**Galectin-3** mRNA and protein levels are increased in PE placental tissue in comparison to normal pregnancy placentas	[100]
	**Galectin-3** is overexpressed in EVT and STB of PE placentas in comparison to normal pregnancy placentas	[94,100,101]
	**Galectin-3** serum levels are higher in PE patients compared to patients with uncomplicated pregnancies	[101,102]
	**Galectin-7** serum levels are higher in women who developed PE in comparison to uncomplicated pregnancies	[16]
	Altered expression of **galectin-9** is detected on peripheral blood lymphocytes in early-onset pre-eclamptic women **Galectin-13** mRNA and protein levels are decreased in placenta tissue in early- and late-onset PE in comparison to healthy pregnancies	[84][103,104]
	**Galectin-13** is overexpressed in STB microvillous membrane in PE compared to healthy controls Low **galectin-13** serum protein level during first trimester, with rapid increase starting with second trimester in women who subsequently develop PE **Galectin-13** in combination with other (bio)markers is a promising tool in PE prediction	[103][62][105]
**Intrauterine growth restriction (IUGR)**	**Galectin-1** low expression in the serum and placenta of pregnant women with IUGR	[106]
	**Galectin-2** expression decreased in male IUGR placentas in all compartments when compared to controls	[15]
	**Galectin-3** expression is significantly higher in cord blood of small-for-gestational-age infants compared to appropriate-for-gestational-age infants	[107]
	**Galectin-3** is significantly downregulated in the EVT of IUGR placentas	[15]
	Placental **galectin-3** expression is downregulated in human pregnancies complicated with IUGR	[67]
	**Galectin-1** and **galectin-3** expression in the EVT is unchanged in IUGR placentas compared with normal controls	[94]
	Significant downregulation of **galectin-4**, **-8** and **-9** in the IUGR trophoblast of male fetuses	[25]
	In IUGR pregnancies with female fetus, **galectin-9** and **galectin-12** are upregulated in the EVT and in endothelial cells in the case of galectin-12; decreased/increased expression in placenta (gender-specific)	[25]
	**Galectin-13** levels, lower than normal, are found in IUGR in the first trimester. In the 2nd and 3rd trimesters, higher than normal concentrations are found in IUGR.	[108]
	Low levels of first-trimester **galectin-13** are associated with preterm birth in women with IUGR	[109]
	Decreased levels of **galectin-13** are not significantly correlated with the studied adverse pregnancy outcomes of IUGR	[110]
	**Galectin-13** expression is strongly decreased in VT and EVT in IUGR-complicated pregnancies of male fetal gender	[15]

EVT—extravillous trophoblast; CTB—cytotrophoblast; STB—syncytiotrophoblast; VT—villous trophoblast; SA—spontaneous abortion; RPL—recurrent pregnancy loss; PPROM—preterm pre-labor rupture of the membranes; SLE—systemic lupus erythematosus; RA—rheumatoid arthritis.

## References

[1] Red-Horse K., Zhou Y., Genbacev O., Prakobphol A., Foulk R., McMaster M., Fisher S.J. (2004). Trophoblast differentiation during embryo implantation and formation of the maternal-fetal interface. J. Clin. Investig..

[2] Blois S.M., Conrad M.L., Freitag N., Barrientos G. (2015). Galectins in angiogenesis: Consequences for gestation. J. Reprod. Immunol..

[3] Blois S.M., Ilarregui J.M., Tometten M., Garcia M., Orsal A.S., Cordo-Russo R., Toscano M.A., Bianco G.A., Kobelt P., Handjiski B. (2007). A pivotal role for galectin-1 in fetomaternal tolerance. Nat. Med..

[4] Than N.G., Romero R., Kim C.J., McGowen M.R., Papp Z., Wildman D.E. (2012). Galectins: Guardians of eutherian pregnancy at the maternal–fetal interface. Trends Endocrinol. Metab..

[5] Johannes L., Jacob R., Leffler H. (2018). Galectins at a glance. J. Cell Sci..

[6] Balogh A., Toth E., Romero R., Parej K., Csala D., Szenasi N.L., Hajdu I., Juhasz K., Kovacs A.F., Meiri H. (2019). Placental Galectins Are Key Players in Regulating the Maternal Adaptive Immune Response. Front. Immunol..

[7] Liu F. (2002). Intracellular functions of galectins. Biochim. Biophys. Acta-Gen. Subj..

[8] Blois S.M., Dveksler G., Vasta G.R., Freitag N., Blanchard V., Barrientos G. (2019). Pregnancy Galectinology: Insights into a Complex Network of Glycan Binding Proteins. Front. Immunol..

[9] Than N.G., Romero R., Goodman M., Weckle A., Xing J., Dong Z., Xu Y., Tarquini F., Szilagyi A., Gal P. (2009). A primate subfamily of galectins expressed at the maternal-fetal interface that promote immune cell death. Proc. Natl. Acad. Sci. USA.

[10] Bevan B.H., Kilpatrick D.C., Liston W.A., Hirabayashi J., Kasai K. (1994). Immunohistochemical localization of a beta-D-galactoside-binding lectin at the human maternofetal interface. Histochem. J..

[11] Vicovac L., Jankovic M., Cuperlovic M. (1998). Galectin-1 and -3 in cells of the first trimester placental bed. Hum. Reprod..

[12] von Wolff M., Wang X., Gabius H.-J., Strowitzki T. (2004). Galectin fingerprinting in human endometrium and decidua during the menstrual cycle and in early gestation. Mol. Hum. Reprod..

[13] Maquoi E., van den Brûle F., Castronovo V., Foidart J. (1997). Changes in the distribution pattern of galectin-1 and galectin-3 in human placenta correlates with the differentiation pathways of trophoblasts. Placenta.

[14] Jeschke U., Hutter S., Heublein S., Vrekoussis T., Andergassen U., Unverdorben L., Papadakis G., Makrigiannakis A. (2013). Expression and function of galectins in the endometrium and at the human feto-maternal interface. Placenta.

[15] Hutter S., Knabl J., Andergassen U., Hofmann S., Kuhn C., Mahner S., Arck P., Jeschke U. (2016). Placental Expression Patterns of Galectin-1, Galectin-2, Galectin-3 and Galectin-13 in Cases of Intrauterine Growth Restriction (IUGR). Int. J. Mol. Sci..

[16] Menkhorst E., Koga K., Van Sinderen M., Dimitriadis E. (2014). Galectin-7 serum levels are altered prior to the onset of pre-eclampsia. Placenta.

[17] Kolundžić N., Bojić-Trbojević Ž., Radojčić L., Petronijević M., Vićovac L. (2011). Galectin-8 is expressed by villous and extravillous trophoblast of the human placenta. Placenta.

[18] Heusschen R., Freitag N., Tirado-González I., Barrientos G., Moschansky P., Muñoz-Fernández R., Leno-Durán E., Klapp B.F., Thijssen V.L.J.L., Blois S.M. (2013). Profiling Lgals9 Splice Variant Expression at the Fetal-Maternal Interface: Implications in Normal and Pathological Human Pregnancy1. Biol. Reprod..

[19] Unverdorben L., Jeschke U., Santoso L., Hofmann S., Kuhn C., Arck P., Hutter S. (2016). Comparative analyses on expression of galectins1-4, 7-10 and 12 in first trimester placenta, decidua and isolated trophoblast cells in vitro. Histol. Histopathol..

[20] Kliman H.J., Sammar M., Grimpel Y.I., Lynch S.K., Milano K.M., Pick E., Bejar J., Arad A., Lee J.J., Meiri H. (2012). Placental Protein 13 and Decidual Zones of Necrosis: An Immunologic Diversion That May be Linked to Preeclampsia. Reprod. Sci..

[21] Than N.G., Pick E., Bellyei S., Szigeti A., Burger O., Berente Z., Janaky T., Boronkai A., Kliman H., Meiri H. (2004). Functional analyses of placental protein13/galectin-13. Eur. J. Biochem..

[22] Chiariotti L., Salvatore P., Frunzio R., Bruni C.B. (2002). Galectin genes: Regulation of expression. Glycoconj. J..

[23] Than N.G., Romero R., Erez O., Weckle A., Tarca A.L., Hotra J., Abbas A., Han Y.M., Kim S.-S., Kusanovic J.P. (2008). Emergence of hormonal and redox regulation of galectin-1 in placental mammals: Implication in maternal-fetal immune tolerance. Proc. Natl. Acad. Sci. USA.

[24] Braun A.E., Muench K.L., Robinson B.G., Wang A., Palmer T.D., Winn V.D. (2021). Examining Sex Differences in the Human Placental Transcriptome during the First Fetal Androgen Peak. Reprod. Sci..

[25] Hutter S., Knabl J., Andergassen U., Mayr D., Hofmann S., Kuhn C., Mahner S., Arck P., Jeschke U. (2015). Fetal gender specific expression of tandem-repeat galectins in placental tissue from normally progressed human pregnancies and intrauterine growth restriction (IUGR). Placenta.

[26] Gitt M.A., Barondes S.H. (1991). Genomic sequence and organization of two members of a human lectin gene family. Biochemistry.

[27] Salvatore P., Contursi C., Benvenuto G., Bruni C.B., Chiariotti L. (1995). Characterization and functional dissection of the galactin-1 gene promoter. FEBS Lett..

[28] Choe Y.S., Shim C., Choi D., Lee C.S., Lee K.-K., Kim K. (1997). Expression of galectin-1 mRNA in the mouse uterus is under the control of ovarian steroids during blastocyst implantation. Mol. Reprod. Dev..

[29] Bojic-Trbojevic Z., Bozic M., Vicovac L. (2008). Steroid hormones modulate galectin-1 in the trophoblast HTR-8/SVneocell line. Arch. Biol. Sci..

[30] Cujic D., Bojic-Trbojevic Z., Tosic N., Pavlovic S., Vicovac L. (2013). Effect of steroids on transcription and secretion of Gal-1 by the human trophoblast cell line in vitro. Arch. Biol. Sci..

[31] Stefanoska I., Jovanović Krivokuća M., Vasilijić S., Ćujić D., Vićovac L. (2013). Prolactin stimulates cell migration and invasion by human trophoblast in vitro. Placenta.

[32] Yang H., Taylor H.S., Lei C., Cheng C., Zhang W. (2011). Hormonal regulation of galectin 3 in trophoblasts and its effects on endometrium. Reprod. Sci..

[33] Yang H., Lei C., Cheng C., Feng Y., Zhang W., Petracco R.G., Sak S. (2012). The Antiapoptotic Effect of Galectin-3 in Human Endometrial Cells under the Regulation of Estrogen and Progesterone1. Biol. Reprod..

[34] Yang H., Lei C.X., Zhang W. (2013). Human chorionic gonadotropin (hCG) regulation of galectin-3 expression in endometrial epithelial cells and endometrial stromal cells. Acta Histochem..

[35] Yang H., Yin J., Ficarrotta K., Hsu S.H., Zhang W., Cheng C. (2016). Aberrant expression and hormonal regulation of Galectin-3 in endometriosis women with infertility. J. Endocrinol. Investig..

[36] Liu F.-T., Rabinovich G.A. (2010). Galectins: Regulators of acute and chronic inflammation. Ann. N. Y. Acad. Sci..

[37] Ramhorst R.E., Giribaldi L., Fraccaroli L., Toscano M.A., Stupirski J.C., Romero M.D., Durand E.S., Rubinstein N., Blaschitz A., Sedlmayr P. (2012). Galectin-1 confers immune privilege to human trophoblast: Implications in recurrent fetal loss. Glycobiology.

[38] Inagaki Y., Sohma Y., Horie H., Nozawa R., Kadoya T. (2000). Oxidized galectin-1 promotes axonal regeneration in peripheral nerves but does not possess lectin properties. Eur. J. Biochem..

[39] Kadoya T., Horie H. (2005). Structural and Functional Studies of Galectin-1: A Novel Axonal Regeneration-Promoting Activity for Oxidized Galectin-1. Curr. Drug Targets.

[40] Sato S., St-Pierre C., Bhaumik P., Nieminen J. (2009). Galectins in innate immunity: Dual functions of host soluble β-galactoside-binding lectins as damage-associated molecular patterns (DAMPs) and as receptors for pathogen-associated molecular patterns (PAMPs). Immunol. Rev..

[41] Kolundžić N., Bojić-Trbojević Ž., Kovačević T., Stefanoska I., Kadoya T., Vićovac L. (2011). Galectin-1 Is Part of Human Trophoblast Invasion Machinery—A Functional Study In Vitro. PLoS ONE.

[42] Kolundžić N., Ćujić D., Abu Rabi T., Bojić-Trbojević Ž., Kadoya T., Vićovac L. (2015). Galectin signature of the choriocarcinoma JAr cells: Galectin-1 as a modulator of invasiveness in vitro. Mol. Reprod. Dev..

[43] Jeschke U., Karsten U., Wiest I., Schulze S., Kuhn C., Friese K., Walzel H. (2006). Binding of galectin-1 (gal-1) to the Thomsen-Friedenreich (TF) antigen on trophoblast cells and inhibition of proliferation of trophoblast tumor cells in vitro by gal-1 or an anti-TF antibody. Histochem. Cell Biol..

[44] Tirado-Gonzalez I., Freitag N., Barrientos G., Shaikly V., Nagaeva O., Strand M., Kjellberg L., Klapp B.F., Mincheva-Nilsson L., Cohen M. (2013). Galectin-1 influences trophoblast immune evasion and emerges as a predictive factor for the outcome of pregnancy. Mol. Hum. Reprod..

[45] Fischer I., Redel S., Hofmann S., Kuhn C., Friese K., Walzel H., Jeschke U. (2010). Stimulation of syncytium formation in vitro in human trophoblast cells by galectin-1. Placenta.

[46] Hutter S., Morales-Prieto D.M., Andergassen U., Tschakert L., Kuhn C., Hofmann S., Markert U.R., Jeschke U. (2016). Gal-1 silenced trophoblast tumor cells (BeWo) show decreased syncytium formation and different miRNA production compared to non-target silenced BeWo cells. Cell Adh. Migr..

[47] Jeschke U., Reimer T., Bergemann C., Wiest I., Schulze S., Friese K., Walzel H. (2004). Binding of galectin-1 (gal-1) on trophoblast cells and inhibition of hormone production of trophoblast tumor cells in vitro by gal-1. Histochem. Cell Biol..

[48] Bojić-Trbojević Ž., Jovanović Krivokuća M., Kolundžić N., Petronijević M., Vrzić-Petronijević S., Golubović S., Vićovac L. (2014). Galectin-1 binds mucin in human trophoblast. Histochem. Cell Biol..

[49] Bojić-Trbojević Z., Jovanović Krivokuća M., Stefanoska I., Kolundzić N., Vilotić A., Kadoya T., Vićovac L. (2018). Integrin β1 is bound to galectin-1 in human trophoblast. J. Biochem..

[50] Zhou Q., Cummings R. (1993). L-14 lectin recognition of laminin and its promotion of in vitro cell adhesion. Arch Biochem Biophys.

[51] Ozeki Y., Matsui T., Yamamoto Y., Funahashi M., Hamako J., Titani K. (1995). Tissue fibronectin is an endogenous ligand for galectin-1. Glycobiology.

[52] Bojić-Trbojević Ž., Jovanović Krivokuća M., Vilotić A., Kolundžić N., Stefanoska I., Zetterberg F., Nilsson U.J., Leffler H., Vićovac L. (2019). Human trophoblast requires galectin-3 for cell migration and invasion. Sci. Rep..

[53] Chen W.-S., Cao Z., Leffler H., Nilsson U.J., Panjwani N. (2017). Galectin-3 Inhibition by a Small-Molecule Inhibitor Reduces Both Pathological Corneal Neovascularization and Fibrosis. Investig. Opthalmol. Vis. Sci..

[54] Freitag N., Tirado-González I., Barrientos G., Cohen M., Daher S., Goldman-Wohl D., Mincheva-Nilsson L., John C.M., Jeschke U., Blois S.M. (2020). The chimera-type galectin-3 is a positive modulator of trophoblast functions with dysregulated expression in gestational diabetes mellitus. Am. J. Reprod. Immunol..

[55] Hu R., Jin H., Zhou S., Yang P., Li X. (2007). Proteomic analysis of hypoxia-induced responses in the syncytialization of human placental cell line BeWo. Placenta.

[56] Menkhorst E.M., Gamage T., Cuman C., Kaitu’U-Lino T.J., Tong S., Dimitriadis E. (2014). Galectin-7 acts as an adhesion molecule during implantation and increased expression is associated with miscarriage. Placenta.

[57] Menkhorst E., Zhou W., Santos L.L., Delforce S., So T., Rainczuk K., Loke H., Syngelaki A., Varshney S., Williamson N. (2020). Galectin-7 Impairs Placentation and Causes Preeclampsia Features in Mice. Hypertension.

[58] Hu X., Zhu Q., Wang Y., Wang L., Li Z., Mor G., Liao A. (2020). Newly characterized decidual Tim-3+ Treg cells are abundant during early pregnancy and driven by IL-27 coordinately with Gal-9 from trophoblasts. Hum. Reprod..

[59] Li Y.-H., Zhou W.-H., Tao Y., Wang S.-C., Jiang Y.-L., Zhang D., Piao H.-L., Fu Q., Li D.-J., Du M.-R. (2016). The Galectin-9/Tim-3 pathway is involved in the regulation of NK cell function at the maternal–fetal interface in early pregnancy. Cell. Mol. Immunol..

[60] Sun J., Yang M., Ban Y., Gao W., Song B., Wang Y., Zhang Y., Shao Q., Kong B., Qu X. (2016). Tim-3 Is Upregulated in NK Cells during Early Pregnancy and Inhibits NK Cytotoxicity toward Trophoblast in Galectin-9 Dependent Pathway. PLoS ONE.

[61] Li M., Peng X., Qian J., Sun F., Chen C., Wang S., Zhang J., Du M. (2021). Galectin-9 regulates HTR8/SVneo function via JNK signaling. Reproduction.

[62] Than N.G., Balogh A., Romero R., Kárpáti É., Erez O., Szilágyi A., Kovalszky I., Sammar M., Gizurarson S., Matkó J. (2014). Placental Protein 13 (PP13)-A Placental Immunoregulatory Galectin Protecting Pregnancy. Front. Immunol..

[63] Vokalova L., Balogh A., Toth E., Van Breda S.V., Schäfer G., Hoesli I., Lapaire O., Hahn S., Than N.G., Rossi S.W. (2020). Placental Protein 13 (Galectin-13) Polarizes Neutrophils Toward an Immune Regulatory Phenotype. Front. Immunol..

[64] Wang M., Xu Y., Wang P., Xu Y., Jin P., Wu Z., Qian Y., Bai L., Dong M. (2021). Galectin-14 Promotes Trophoblast Migration and Invasion by Upregulating the Expression of MMP-9 and N-Cadherin. Front. Cell Dev. Biol..

[65] Liu A.-X., Jin F., Zhang W.-W., Zhou T.-H., Zhou C.-Y., Yao W.-M., Qian Y.-L., Huang H.-F. (2006). Proteomic Analysis on the Alteration of Protein Expression in the Placental Villous Tissue of Early Pregnancy Loss. Biol. Reprod..

[66] Unverdorben L., Haufe T., Santoso L., Hofmann S., Jeschke U., Hutter S. (2016). Prototype and Chimera-Type Galectins in Placentas with Spontaneous and Recurrent Miscarriages. Int. J. Mol. Sci..

[67] Freitag N., Tirado-Gonzalez I., Barrientos G., Powell K.L., Boehm-Sturm P., Koch S.P., Hecher K., Staff A.C., Arck P.C., Diemert A. (2020). Galectin-3 deficiency in pregnancy increases the risk of fetal growth restriction (FGR) via placental insufficiency. Cell Death Dis..

[68] Xiao Q., Zeng F., Tang G., Lei C., Zou X., Liu X., Peng B., Qin S., Li H. (2019). Expression of galectin-3 and apoptosis in placental villi from patients with missed abortion during early pregnancy. Exp. Ther. Med..

[69] Gao L., Fang A. (2014). Expression and Influence of Galectin-3 on Missed Abortion. J. Reprod. Contracept..

[70] Blois S.M., Gueuvoghlanian-Silva B.Y., Tirado-Gonzalez I., Torloni M.R., Freitag N., Mattar R., Conrad M.L., Unverdorben L., Barrientos G., Knabl J. (2014). Getting too sweet: Galectin-1 dysregulation in gestational diabetes mellitus. Mol. Hum. Reprod..

[71] Hepp P., Unverdorben L., Hutter S., Kuhn C., Ditsch N., Groß E., Mahner S., Jeschke U., Knabl J., Heidegger H.H. (2020). Placental Galectin-2 Expression in Gestational Diabetes: A Systematic, Histological Analysis. Int. J. Mol. Sci..

[72] Talmor-Barkan Y., Chezar-Azerrad C., Kruchin B., Leshem-Lev D., Levi A., Hadar E., Kornowski R., Tenenbaum-Gavish K., Porter A. (2020). Elevated galectin-3 in women with gestational diabetes mellitus, a new surrogate for cardiovascular disease in women. PLoS ONE.

[73] Zhang Z., Kang X., Guo Y., Zhang J., Xie J., Shao S., Xiang Y., Chen G., Yu X. (2021). Association of circulating galectin-3 with gestational diabetes mellitus, progesterone, and insulin resistance. J. Diabetes.

[74] Heusler I., Biron-Shental T., Farladansky-Gershnabel S., Pasternak Y., Kidron D., Vulih-Shuitsman I., Einbinder Y., Cohen-Hagai K., Benchetrit S., Zitman-Gal T. (2021). Enhanced expression of Galectin-3 in gestational diabetes. Nutr. Metab. Cardiovasc. Dis..

[75] Boutsikou T., Giotaki M., Boutsikou M., Briana D.D., Baka S., Piatopoulou D., Hassiakos D., Gourgiotis D., Malamitsi-Puchner A. (2015). Cord blood galectin-1 and -3 concentrations in term pregnancies with normal restricted and increased fetal growth. J. Perinat. Med..

[76] Unverdorben L., Hüttenbrenner R., Knabl J., Jeschke U., Hutter S. (2015). Galectin-13/PP-13 expression in term placentas of gestational diabetes mellitus pregnancies. Placenta.

[77] Zhao B., Han X., Meng Q., Luo Q. (2018). Early second trimester maternal serum markers in the prediction of gestational diabetes mellitus. J. Diabetes Investig..

[78] Than N.G., Erez O., Wildman D.E., Tarca A.L., Edwin S.S., Abbas A., Hotra J., Kusanovic J.P., Gotsch F., Hassan S.S. (2008). Severe preeclampsia is characterized by increased placental expression of galectin-1. J. Matern. Neonatal Med..

[79] Stefanoska I., Tadić J., Vilotić A., Jovanović Krivokuća M., Abu Rabi T., Vićovac L. (2017). Histological chorioamnionitis in preterm prelabor rupture of the membranes is associated with increased expression of galectin-3 by amniotic epithelium. J. Matern. Neonatal Med..

[80] Offenbacher S., Katz V., Fertik G., Collins J., Boyd D., Maynor G., McKaig R., Beck J. (1996). Periodontal Infection as a Possible Risk Factor for Preterm Low Birth Weight. J. Periodontol..

[81] Bélanger M., Reyes L., von Deneen K., Reinhard M.K., Progulske-Fox A., Brown M.B. (2008). Colonization of maternal and fetal tissues by Porphyromonas gingivalis is strain-dependent in a rodent animal model. Am. J. Obstet. Gynecol..

[82] Leavy O. (2008). TIM3: Dual role in immunity. Nat. Rev. Immunol..

[83] Chabtini L., Mfarrej B., Mounayar M., Zhu B., Batal I., Dakle P.J., Smith B.D., Boenisch O., Najafian N., Akiba H. (2013). TIM-3 Regulates Innate Immune Cells To Induce Fetomaternal Tolerance. J. Immunol..

[84] Miko E., Meggyes M., Bogar B., Schmitz N., Barakonyi A., Varnagy A., Farkas B., Tamas P., Bodis J., Szekeres-Bartho J. (2013). Involvement of Galectin-9/TIM-3 Pathway in the Systemic Inflammatory Response in Early-Onset Preeclampsia. PLoS ONE.

[85] Li Z.-H., Wang L.-L., Liu H., Muyayalo K.P., Huang X.-B., Mor G., Liao A.-H. (2019). Galectin-9 Alleviates LPS-Induced Preeclampsia-Like Impairment in Rats via Switching Decidual Macrophage Polarization to M2 Subtype. Front. Immunol..

[86] Lutomski D., Joubert-Caron R., Lefebure C., Salama J., Belin C., Bladier D., Michel C. (1997). Anti-galectin-1 autoantibodies in serum of patients with neurological diseases. Clin. Chim. Acta.

[87] Romero M.D., Muin~o J.C., Bianco G.A., Ferrero M., Juarez C.P., Luna J.D., Rabinovich G.A. (2006). Circulating Anti-galectin-1 Antibodies Are Associated with the Severity of Ocular Disease in Autoimmune and Infectious Uveitis. Investig. Opthalmol. Vis. Sci..

[88] Montiel J., Monsiváis-Urenda A., Figueroa-Vega N., Moctezuma J., Burgos-Vargas R., González-Amaro R., Rosenstein Y. (2010). Anti-CD43 and anti-galectin-1 autoantibodies in patients with systemic lupus erythematosus. Scand. J. Rheumatol..

[89] Sarter K., Janko C., Andre S., Munoz L.E., Schorn C., Winkler S., Rech J., Kaltner H., Lorenz H.-M., Schiller M. (2013). Autoantibodies against galectins are associated with antiphospholipid syndrome in patients with systemic lupus erythematosus. Glycobiology.

[90] Kang E., Moon K., Lee E., Lee Y., Lee E., Ahn C., Song Y. (2009). Renal expression of galectin-3 in systemic lupus erythematosus patients with nephritis. Lupus.

[91] Shi Z., Tan G., Meng Z., Yu M., Li K., Yin J., Wei K., Luo Y., Jia S., Zhang S. (2015). Association of Anti-Acidic Ribosomal Protein P0 and Anti-Galectin 3 Antibodies With the Development of Skin Lesions in Systemic Lupus Erythematosus. Arthritis Rheumatol..

[92] Lim Y., Lee D.-Y., Lee S., Park S.-Y., Kim J., Cho B., Lee H., Kim H.-Y., Lee E., Song Y.W. (2002). Identification of autoantibodies associated with systemic lupus erythematosus. Biochem. Biophys. Res. Commun..

[93] Massardo L., Metz C., Pardo E., Mezzano V., Babul M., Jarpa E., Guzmán A., André S., Kaltner H., Gabius H. (2009). Autoantibodies against galectin-8: Their specificity, association with lymphopenia in systemic lupus erythematosus and detection in rheumatoid arthritis and acute inflammation. Lupus.

[94] Jeschke U., Mayr D., Schiessl B., Mylonas I., Schulze S., Kuhn C., Friese K., Walzel H. (2007). Expression of Galectin-1, -3 (gal-1, gal-3) and the Thomsen–Friedenreich (TF) Antigen in Normal, IUGR, Preeclamptic and HELLP Placentas. Placenta.

[95] Alese O.M., Moodley J., Naicker T. (2020). The role of Galectin-1 in HIV associated preeclampsia. Eur. J. Obstet. Gynecol. Reprod. Biol..

[96] Molvarec A., Blois S.M., Stenczer B., Toldi G., Tirado-Gonzalez I., Ito M., Shima T., Yoneda S., Vásárhelyi B., Rigó J. (2011). Peripheral blood galectin-1-expressing T and natural killer cells in normal pregnancy and preeclampsia. Clin. Immunol..

[97] Freitag N., Tirado-Gonzalez I., Barrientos G., Herse F., Thijssen V.L.J.L., Weedon-Fekjaer S.M., Schulz H., Wallukat G., Klapp B.F., Nevers T. (2013). Interfering with Gal-1-mediated angiogenesis contributes to the pathogenesis of preeclampsia. Proc. Natl. Acad. Sci. USA.

[98] Hirashima C., Ohkuchi A., Nagayama S., Suzuki H., Takahashi K., Ogoyama M., Takahashi H., Shirasuna K., Matsubara S. (2018). Galectin-1 as a novel risk factor for both gestational hypertension and preeclampsia, specifially its expression at a low level in the second trimester and a high level after onset. Hypertens. Res..

[99] Hutter S., Martin N., von Schönfeldt V., Messner J., Kuhn C., Hofmann S., Andergassen U., Knabl J., Jeschke U. (2015). Galectin 2 (gal-2) expression is downregulated on protein and mRNA level in placentas of preeclamptic (PE) patients. Placenta.

[100] Ruikar K., Aithal M., Shetty P., Dinesh U.S., Bargale A., Sadashiv R., Sarathkumar E., Khode V., Desai R., Patil P. (2021). Placental Expression and Relative Role of Anti-inflammatory Annexin A1 and Animal Lectin Galectin-3 in the Pathogenesis of Preeclampsia. Indian J. Clin. Biochem..

[101] Pankiewicz K., Szczerba E., Fijalkowska A., Szamotulska K., Szewczyk G., Issat T., Maciejewski T. (2020). The association between serum galectin-3 level and its placental production in patients with preeclampsia. J. Physiol. Pharmacol..

[102] Sattar Taha A., Zahraei Z., Al-Hakeim H. (2020). Serum apelin and galectin-3 in preeclampsia in Iraq. Hypertens. Pregnancy.

[103] Than N.G., Abdul Rahman O., Magenheim R., Nagy B., Fule T., Hargitai B., Sammar M., Hupuczi P., Tarca A.L., Szabo G. (2008). Placental protein 13 (galectin-13) has decreased placental expression but increased shedding and maternal serum concentrations in patients presenting with preterm pre-eclampsia and HELLP syndrome. Virchows Arch..

[104] Sammar M., Nisemblat S., Fleischfarb Z., Golan A., Sadan O., Meiri H., Huppertz B., Gonen R. (2011). Placenta-bound and Body Fluid PP13 and its mRNA in Normal Pregnancy Compared to Preeclampsia, HELLP and Preterm Delivery. Placenta.

[105] Khalil A., Cowans N.J., Spencer K., Goichman S., Meiri H., Harrington K. (2010). First trimester markers for the prediction of pre-eclampsia in women with a priori high risk. Ultrasound Obstet. Gynecol..

[106] Jin X.-X., Ying X., Dong M.-Y. (2021). Galectin-1 expression in the serum and placenta of pregnant women with fetal growth restriction and its significance. BMC Pregnancy Childbirth.

[107] Demmert M., Faust K., Bohlmann M.K., Tröger B., Göpel W., Herting E., Härtel C. (2012). Galectin-3 in cord blood of term and preterm infants. Clin. Exp. Immunol..

[108] Burger O., Pick E., Zwickel J., Klayman M., Meiri H., Slotky R., Mandel S., Rabinovitch L., Paltieli Y., Admon A. (2004). Placental protein 13 (PP-13): Effects on cultured trophoblasts, and its detection in human body fluids in normal and pathological pregnancies. Placenta.

[109] Chafetz I., Kuhnreich I., Sammar M., Tal Y., Gibor Y., Meiri H., Cuckle H., Wolf M. (2007). First-trimester placental protein 13 screening for preeclampsia and intrauterine growth restriction. Am. J. Obstet. Gynecol..

[110] Cowans N.J., Spencer K., Meiri H. (2008). First-trimester maternal placental protein 13 levels in pregnancies resulting in adverse outcomes. Prenat. Diagn..

[111] Frick A.P. (2021). Advanced maternal age and adverse pregnancy outcomes. Best Pract. Res. Clin. Obstet. Gynaecol..

[112] Ilekis J.V., Tsilou E., Fisher S., Abrahams V.M., Soares M.J., Cross J.C., Zamudio S., Illsley N.P., Myatt L., Colvis C. (2016). Placental origins of adverse pregnancy outcomes: Potential molecular targets: An Executive Workshop Summary of the Eunice Kennedy Shriver National Institute of Child Health and Human Development. Am. J. Obstet. Gynecol..

[113] Wu M., Liu P., Cheng L. (2015). Galectin-1 reduction and changes in T regulatory cells may play crucial roles in patients with unexplained recurrent spontaneous abortion. Int. J. Clin. Exp. Pathol..

[114] Mack L.R., Tomich P.G. (2017). Gestational Diabetes. Obstet. Gynecol. Clin. N. Am..

[115] Ben-Haroush A., Yogev Y., Hod M. (2004). Epidemiology of gestational diabetes mellitus and its association with Type 2 diabetes. Diabet. Med..

[116] Desoye G., Hauguel-de Mouzon S. (2007). The Human Placenta in Gestational Diabetes Mellitus: The insulin and cytokine network. Diabetes Care.

[117] Crowther C.A., Hiller J.E., Moss J.R., McPhee A.J., Jeffries W.S., Robinson J.S. (2005). Effect of Treatment of Gestational Diabetes Mellitus on Pregnancy Outcomes. N. Engl. J. Med..

[118] Li P., Liu S., Lu M., Bandyopadhyay G., Oh D., Imamura T., Johnson A.M.F., Sears D., Shen Z., Cui B. (2016). Hematopoietic-Derived Galectin-3 Causes Cellular and Systemic Insulin Resistance. Cell.

[119] Kuc S., Wortelboer E., Koster M., de Valk H., Schielen P., Visser G. (2011). Prediction of macrosomia at birth in type-1 and 2 diabetic pregnancies with biomarkers of early placentation. BJOG An Int. J. Obstet. Gynaecol..

[120] Rabinovich G.A., Gruppi A. (2005). Galectins as immunoregulators during infectious processes: From microbial invasion to the resolution of the disease. Parasite Immunol..

[121] Rabinovich G.A., Liu F.-T., Hirashima M., Anderson A. (2007). An Emerging Role for Galectins in Tuning the Immune Response: Lessons from Experimental Models of Inflammatory Disease, Autoimmunity and Cancer. Scand. J. Immunol..

[122] Blidner A.G., Rabinovich G.A. (2013). ‘Sweetening’ Pregnancy: Galectins at the Fetomaternal Interface. Am. J. Reprod. Immunol..

[123] Romero R., Gotsch F., Pineles B., Kusanovic J.P. (2008). Inflammation in Pregnancy: Its Roles in Reproductive Physiology, Obstetrical Complications, and Fetal Injury. Nutr. Rev..

[124] Kourtis A.P., Read J.S., Jamieson D.J. (2014). Pregnancy and Infection. N. Engl. J. Med..

[125] Cotechini T., Graham C.H. (2015). Aberrant maternal inflammation as a cause of pregnancy complications: A potential therapeutic target?. Placenta.

[126] Cappelletti M., Della Bella S., Ferrazzi E., Mavilio D., Divanovic S. (2016). Inflammation and preterm birth. J. Leukoc. Biol..

[127] Fuertes M.B., Molinero L.L., Toscano M.A., Ilarregui J.M., Rubinstein N., Fainboim L., Zwirner N.W., Rabinovich G.A. (2004). Regulated expression of galectin-1 during T-cell activation involves Lck and Fyn kinases and signaling through MEK1/ERK, p38 MAP kinase and p70 ^S6^ kinase. Mol. Cell. Biochem..

[128] Than N.G., Wildman D.E., Erez O., Edwin S.S., Espinoza J., Kim C.J., Han Y.M., Mazaki-Tovi S., Kusanovic J.P., Hassan S. (2006). Trophoblast, Galectin-1 and pre-eclampsia. Am. J. Obstet. Gynecol..

[129] Bonney E.A. (2007). Preeclampsia: A view through the danger model. J. Reprod. Immunol..

[130] Kim Y.M., Romero R., Oh S.Y., Kim C.J., Kilburn B.A., Armant D.R., Nien J.K., Gomez R., Mazor M., Saito S. (2005). Toll-like receptor 4: A potential link between “danger signals,” the innate immune system, and preeclampsia?. Am. J. Obstet. Gynecol..

[131] Balogh A., Pozsgay J., Matkó J., Dong Z., Kim C.J., Várkonyi T., Sammar M., Rigó J., Meiri H., Romero R. (2011). Placental protein 13 (PP13/galectin-13) undergoes lipid raft-associated subcellular redistribution in the syncytiotrophoblast in preterm preeclampsia and HELLP syndrome. Am. J. Obstet. Gynecol..

[132] Burton G.J., Yung H.-W., Cindrova-Davies T., Charnock-Jones D.S. (2009). Placental Endoplasmic Reticulum Stress and Oxidative Stress in the Pathophysiology of Unexplained Intrauterine Growth Restriction and Early Onset Preeclampsia. Placenta.

[133] Giordanengo L., Gea S., Barbieri G., Rabinovich G.A. (2001). Anti-galectin-1 autoantibodies in human *Trypanosoma cruzi* infection: Differential expression of this *β*-galactoside-binding protein in cardiac Chagas’ disease. Clin. Exp. Immunol..

[134] Burton G., Redman C., Roberts J., Moffett A. (2019). Pre-eclampsia: Pathophysiology and clinical implications. BMJ.

[135] Robillard P., Dekker G., Scioscia M., Bonsante F., Iacobelli S., Boukerrou M., Hulsey T.C. (2020). Validation of the 34-week gestation as definition of late onset preeclampsia: Testing different cutoffs from 30 to 37 weeks on a population-based cohort of 1700 preeclamptics. Acta Obstet. Gynecol. Scand..

[136] Xu B., Shanmugalingam R., Chau K., Makris A., Hennessy A. (2020). Galectin-1–Related Modulation of Trophoblast Endothelial Interactions by Integrins α1 and β1. Reprod. Sci..

[137] Deng Q., Chen Y., Yin N., Shan N., Luo X., Yuan Y., Luo X., Liu Y., Liu X., Qi H. (2017). The Role of MGAT5 in Human Umbilical Vein Endothelial Cells. Reprod. Sci..

[138] Charkiewicz K., Goscik J., Raba G., Laudanski P. (2021). Syndecan 4, galectin 2, and death receptor 3 (DR3) as novel proteins in pathophysiology of preeclampsia. J. Matern. Neonatal Med..

[139] Nikolov A., Popovski N., Blazhev A. (2020). Serum Galectin-3 Levels Are Unlikely to Be a Useful Predictive Marker for Early-onset Preeclampsia Development. Prague Med. Rep..

[140] Sekizawa A., Purwosunu Y., Yoshimura S., Nakamura M., Shimizu H., Okai T., Rizzo N., Farina A. (2009). PP13 mRNA Expression in Trophoblasts From Preeclamptic Placentas. Reprod. Sci..

[141] Farina A., Zucchini C., Sekizawa A., Purwosunu Y., de Sanctis P., Santarsiero G., Rizzo N., Morano D., Okai T. (2010). Performance of messenger RNAs circulating in maternal blood in the prediction of preeclampsia at 10–14 weeks. Am. J. Obstet. Gynecol..

[142] Huppertz B., Meiri H., Gizurarson S., Osol G., Sammar M. (2013). Placental protein 13 (PP13): A new biological target shifting individualized risk assessment to personalized drug design combating pre-eclampsia. Hum. Reprod. Update.

[143] Nicolaides K.H., Bindra R., Turan O.M., Chefetz I., Sammar M., Meiri H., Tal J., Cuckle H.S. (2005). A novel approach to first-trimester screening for early pre-eclampsia combining serum PP-13 and Doppler ultrasound. Ultrasound Obstet. Gynecol..

[144] Wortelboer E., Koster M., Cuckle H., Stoutenbeek P., Schielen P., Visser G. (2010). First-trimester placental protein 13 and placental growth factor: Markers for identification of women destined to develop early-onset pre-eclampsia. BJOG Int. J. Obstet. Gynaecol..

[145] Cuckle H.S. (2011). Screening for Pre-eclampsia–Lessons from Aneuploidy Screening. Placenta.

[146] Mandruzzato G., Antsaklis A., Botet F., Chervenak F.A., Figueras F., Grunebaum A., Puerto B., Skupski D., Stanojevic M. (2008). Intrauterine restriction (IUGR). J. Perinat. Med..

[147] Brodsky D., Christou H. (2004). Current Concepts in Intrauterine Growth Restriction. J. Intensive Care Med..

[148] Sharma D., Shastri S., Farahbakhsh N., Sharma P. (2016). Intrauterine growth restriction–part 1. J. Matern. Neonatal Med..

[149] Pankiewicz K., Maciejewski T. (2017). Perinatal mortality and morbidity of growth restricted fetuses and newborns (own experience)-first report. Dev. Period Med..

[150] Moros G., Boutsikou T., Fotakis C., Iliodromiti Z., Sokou R., Katsila T., Xanthos T., Iacovidou N., Zoumpoulakis P. (2021). Insights into intrauterine growth restriction based on maternal and umbilical cord blood metabolomics. Sci. Rep..

[151] Albu A.R., Anca A.F., Horhoianu V.V., Horhoianu I.A. (2014). Predictive factors for intrauterine growth restriction. J. Med. Life.

[152] Sammar M., Drobnjak T., Mandala M., Gizurarson S., Huppertz B., Meiri H. (2019). Galectin 13 (PP13) Facilitates Remodeling and Structural Stabilization of Maternal Vessels during Pregnancy. Int. J. Mol. Sci..

[153] Scifres C.M., Nelson D.M. (2009). Intrauterine growth restriction, human placental development and trophoblast cell death. J. Physiol..

